# Agents of swimmer’s itch—dangerous minority in the Digenea invasion of Lymnaeidae in water bodies and the first report of *Trichobilharzia regenti* in Poland

**DOI:** 10.1007/s00436-018-6068-3

**Published:** 2018-09-13

**Authors:** Anna Marszewska, Tomasz Strzała, Anna Cichy, Grażyna B. Dąbrowska, Elżbieta Żbikowska

**Affiliations:** 1 Department of Invertebrate Zoology, Faculty of Biology and Environment Protection, Nicolaus Copernicus University in Toruń, Toruń, Poland; 2 Department of Genetics, Faculty of Biology and Animal Science, Wrocław University of Environmental and Life Sciences, Wrocław, Poland; 3 Department of Genetics, Faculty of Biology and Environment Protection, Nicolaus Copernicus University in Toruń, Toruń, Poland

**Keywords:** Trichobilharzia, Lymnaeidae, Molecular identification, Prevalence, Seasonality, Shell size

## Abstract

*Trichobilharzia* spp. have been identified as a causative agent of swimmers’ itch, a skin disease provoked by contact with these digenean trematodes in water. These parasites have developed a number of strategies to invade vertebrates. Since we have little understanding of the behavior of these parasites inside the human body, the monitoring of their invasion in snail host populations is highly recommended. In our research, lymnaeid snails were collected from several Polish lakes for two vegetation seasons. The prevalence of bird schistosomes in snail host populations was significantly lower than that of other digenean species. We were the first to detect the presence of the snails emitted *Trichobilharzia regenti* (potentially the most dangerous nasal schistosome) in Poland. In addition, by sequencing partial rDNA genes, we confirmed the presence of the snails positive with *Trichobilharzia szidati* in Polish water bodies, showing that swimmer’s itch is more frequent during summer months and that large snails are more often infected with bird schistosomes than small ones.

**Electronic supplementary material:**

The online version of this article (10.1007/s00436-018-6068-3) contains supplementary material, which is available to authorized users.

## Introduction

Digenea include parasites with a complex life cycle. Generally, they use snails as their first intermediate hosts, and vertebrates, as final (Cheng 1986; Cichy et al. 2011). Many digenetic trematodes pose threat to animal and human health (Cheng 1986). These parasites include bird schistosomes, whose invasion on humans has been recently reported in many countries (Rao et al. 2007; Valdovinos and Balboa 2008; Gohardehi et al. 2013; Marie et al. 2015; Marszewska et al. 2016; Caron et al. 2017). The disease caused by cercariae of these trematodes occurs globally and is considered to be re-emerging (Kolářová et al. 2010).

The first intermediate hosts of widely spread *Trichobilharzia* spp. include freshwater snail species of the family Lymnaeidae (Horák et al. 2002). Their final hosts include waterfowl of the families Ardeidae, Podicipedidae, Ciconiidae, and Anatidae (Sulgostowska and Czaplińska 1987; Rudolfová et al. 2007; Jouet et al. 2009). The specificity of bird schistosomes is much lower in relation to the final than to the intermediate host. According to Horák et al. (2002), the same species can develop and sexually reproduce in representatives of different bird families. Cercariae of the parasites are released from snails and seek their final vertebrate hosts using chemoreceptors. They respond to external stimuli (e.g., shadow) and signals from a potential host (e.g., fatty acids) (Horák et al. 2008). When the search is complete, larvae penetrate the host through the skin. In this process, the products of parasite’s penetration glands are activated (Horák et al. 1997; Mikeš et al. 2005). The similarity between some lipid components in the integument of humans and birds makes the parasites attack people wading, swimming, or working in water (Haas and van de Roemer 1998). The consequence of such invasions is a skin disease known as swimmers’ itch (Kolářová et al. 2010), whose first symptoms appear within 2 h after the exposure to cercariae. Within the next 2 days, the symptoms worsen. The rash, which initially causes only redness and itching, develops into small, red bumps (Żbikowska et al. 2002). The severity of symptoms may vary, depending on the number of parasites attacking the skin (Żbikowska 2003). The affected area is warm, swollen, and painful. A pricking, tingling, and sometimes burning sensation leads to discomfort and even insomnia (Żbikowska et al. 2002). The condition may be accompanied by other symptoms including swollen lymph nodes, diarrhea, nausea, or fever (Horák et al. 2002; Żbikowska et al. 2002). Occasionally, anaphylactic shocks or respiratory system disorders may also be observed (Bayssade–Dufour et al. 2001). The disease intensity depends on the individual susceptibility of the host (Kolářová et al. 2013a).

The swimmer’s itch is a recurrent disease listed also in Poland. Therefore, there are several reasons for monitoring the prevalence of these parasites *Trichobilharzia* spp. in snail populations: (i) the abundance of snails releasing cercariae of *Trichobilharzia* spp., (ii) the abundance of water birds, (iii) frequent cases of itchy rashes (with a range of symptoms), especially in children, (iv) the lack of data on the behavior of cercariae invading humans through the skin. It is recommended that monitoring should be conducted in two ways: (i) using molecular method to identify particularly dangerous nasal schistosomes and (ii) conducting environmental inspection of recreational water bodies. The present study was aimed at investigating the diversity of Trichobilharzia species in Poland and highlighting the risk of swimmer’s itch based on the spread of bird schistosomes in intermediate snail host populations (prevalence and seasonal fluctuations of parasite invasion, shell size of snail hosts) and in comparison with overall risk of Digenea invasion.

## Material and methods

### Field sampling

Lymnaeid snails (intermediate hosts of *Tricholilharzia* spp.), namely *Lymnaea stagnalis*, *Radix* spp., and *Stagnicola palustris* were collected monthly from May to September in 2016 and 2017. In 2016, they were collected from seven lakes of central and northern Poland: Głuszyńskie (52° 29′ 8″ N, 18° 38′ 13″ E), Ostrowąskie (52° 49′ 46″ N, 18° 42′ 3″ E), Służewskie (52° 51′ 14″ N, 18° 38′ 38″ E), Skulskie (52° 28′ 0″ N, 18° 19′ 18″ E) (Kuyavian-Pomeranian Voivodeship), Skulska Wieś (52° 28′ 58″ N, 18° 19′ 34″ E) (Greater Poland Voivodeship), Szymbarskie (53° 36′ 52″ N, 19° 30′ 39″ E) (Warmian-MasurianVoivodeship), and Wodna Dolina (Water Valley) (54° 10′ 45″ N, 16° 11′ 15″ E) (West Pomeranian Voivodeship), while in 2017, from three lakes: Głuszyńskie, Skulskie, and Skulska Wieś. Research sites were selected based on a combination of factors such as preliminary parasitological tests of snails in 2015, the presence of waterfowl, and previous reports of swimmer’s itch episodes.

The snails, collected from the littoral zone (depth of ca. 0.5–1.5 m) of each lake by two researchers within the span of 1 h and under stable weather conditions, were transported to the laboratory in containers with lake water and examined for Digenea invasion.

### Snail/cercaria examination

Piechocki’s and Wawrzyniak-Wydrowska’s (2016) and Jackiewicz’s (2000) keys were used for morphological and anatomical identification of snails. Shell sizes (shell lengths) were measured using an electronic caliper (accuracy of 0.1 mm). Snails were placed individually in beakers with a small amount of conditioned tap water and exposed to artificial light for 3 h to stimulate the release of cercariae. Larval species were preliminarily determined using a light microscope (Primostar Carl Zeiss) and available keys, descriptions, and pictures from numerous publications on these parasites (Combes 1980; Našincová 1992; Faltýnková et al. 2007, 2008; Cichy and Żbikowska 2016). When no cercariae were released into water, an autopsy of snail hepatopancreas and gonads was carried out. The digenetic species were identified from fully developed cercariae. Morphologically classified larvae of bird schistosomes were subjected to molecular identification.

### DNA extraction, PCR amplification, sequencing, and phylogenetic analyses

The suspension of bird schistosome cercariae was centrifuged. The isolated larvae were preserved in ethanol (96%) and frozen (at − 20 °C) for subsequent molecular identification (Jouet et al. 2008). Several dozens of cercariae were used for DNA extraction. Prior to DNA extraction, cercariae were centrifuged at 5000*g* for 5 min and washed three times in PBS buffer (pH 7.4). Total genomic DNA was isolated with Sherlock AX (A&A Biotechnology, Gdynia, Poland), according to the manufacturer’s instruction. The quality and quantity of the isolated DNA was assessed in gel electrophoresis (1% agarose gel). The partial nuclear ribosomal 28S rDNA (D1–D3) gene (28SrDNA) of cercariae released from *Radix* spp. was amplified using the forward primer DLS1 (5′-ACCCGCTGAACTTAAGCATATCACTAAGC-3′) (Laskowski and Rocka 2014) and the reverse primer 1500R (5′-GCTATCCTGAGGGAAACTTCG-3′) (Tkach et al. 2003). A fragment of the ribosomal DNA of bird schistosomes invading *L. stagnalis*, spanning the sequences of internal transcribed spacers 1, 2, and 5.8S (ITS), was amplified using the forward primer its5Trem (5′-GGAAGTAAAAGTCGTAACAAGG-3′) and the reverse primer its4Trem (5′-TCCTCCGCTTATTGATATGC-3′) (Dvorák et al. 2002) according to PCR conditions described by Dvorák et al. (2002).The amplified products were purified with Clean-Up (A&A Biotechnology, Gdynia, Poland) according to the producer’s manual. DNA product sequencing in both directions was carried out by Genomed S. A., Warsaw. From the obtained genetic material, one sample from each research site for each snail species was used for sequencing. Furthermore, species membership of the analyzed individuals was identified with phylogenetic approach within two datasets (28rDNA and ITS). Newly sequenced haplotypes, along with homological DNA sequences from GenBank (Supplementary Material 1, 2), were first aligned using Muscle algorithm (Edgar 2004) implemented in Seaview software (Gouy et al. 2010). After the alignment, the sequences were cut to obtain a uniform block of sequences and a model of nucleotide substitution was chosen for each dataset using jModelTest 2.1.10 (Darriba et al. 2012). Next, both datasets were analyzed with MrBayes 3.2.6 (Ronquist et al. 2012) using GTR +G as the best-fit model. Two independent runs of four chains starting from different random trees were used. The trees were sampled every 100th generation for 25,000,000 generations of Markov chain steps and all trees making up the final tree were probed when the average standard deviation between the runs was much lower than 0.01.

### Statistical analysis

Seasonal infection fluctuations were calculated and the results were analyzed using Friedman rank test followed by post-hoc Wilcoxon signed-rank test. A chi-square test of contingency table was used to determine statistical differences in the number of snails infected with bird schistosomes and uninfected ones between four shell size classes. The same analysis was used to compare the numbers of snails infected and uninfected with Digenea. Next, post hoc test based on standardized residuals was used. Standardized residuals presented the degree to which an observed value deviates from the expected value in terms of a *z* score (Sidanius et al. 2008). A standardized residual (SR) of plus or minus 1.96 presented a significant deviation from 0 at the *p* = 0.05 level (Sidanius et al. 2008). Statistical analysis was prepared based only on data on bird schistosomes from *L. stagnalis*. The term prevalence was used for the description of one snail species invaded by one parasite species.

## Results

### Larval trematode infection in Lymnaeidae

**Fig. 1:**
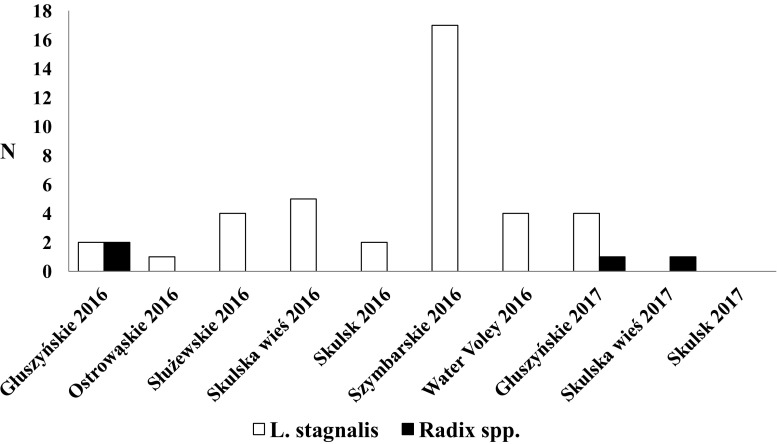
The number of Lymnaeidae infected with bird schistosomes at all research sites

We collected a total of 3456 snails (2484 in the first and 972 in the second year of study): 2325 individuals of *L. stagnalis*, 890 *Radix* spp., and 240 *S. palustris*. Over 30% of the collected Lymneaidae were infected with Digenea. The infection was most frequent among *L. stagnalis* (36.34%) (Table S1), followed by *S. palustris* (21.25%) (Table S2), and *Radix* spp. (18.08%) (Table S3). The following species were most frequent in *L. stagnalis*: *Diplostomum pseudospathaceum* Niewiadomska, 1984 (in 27.3% of infected snails)*, Opisthioglyphe ranae* (Frolich, 1791) (20%), *Plagiorchis elegans* (Rudolphi, 1802) (12.5%), and *Echinoparyphium aconiatum* (Dietz, 1909) (11.5%). *Radix* spp. were most frequently invaded by *O. ranae* (19.9%), *Cotylurus* sp. (13.7%), *Echinoparyphium recurvatum* (Linstow, 1873) (12.4%), and *P. elegans* (8.1%), while *S. palustris,* by *D. pseudospathaceum* (27.5%), *E. aconiatum* (9.8%)*, Hypoderaeum conoideum* (Bloch, 1782) (9.8%), *Molinella anceps* (Molin, 1859) (9.8%), and *O. ranae* (9.8%). Bird schistosomes were recorded in 1.24% of all examined snails. *L. stagnalis* were the most common host of bird schistosomes (1.68%), followed by *Radix* spp. (0.44%). *S. palustris* were not infected with these parasites (Fig. 1). *L. stagnalis* were found in all seven studied lakes, while *Radix* spp., only in two (Fig. 1).

### Molecular analyses of cercariae of bird schistosomes

**Fig. 2:**
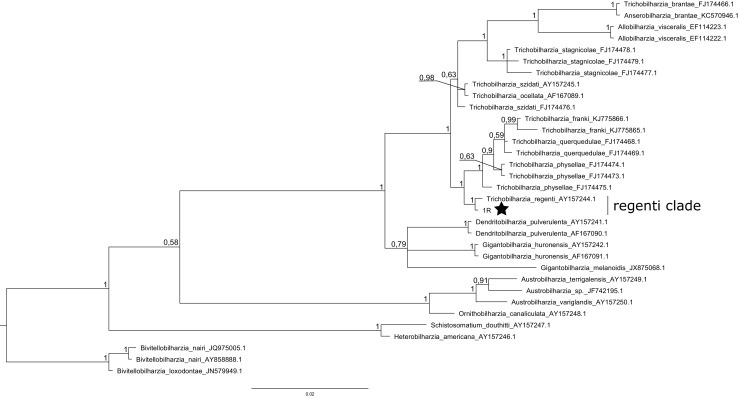
Bayesian phylogenetic tree of dataset X consisted of 33 sequences of bird schistosomes. Three sequences from representatives of *Bivitellobriharzia nairi* were used as an outgroup. Numbers along the nodes are posterior probability of the node. DNA sequence revealed in this study is marked with a star

**Fig. 3:**
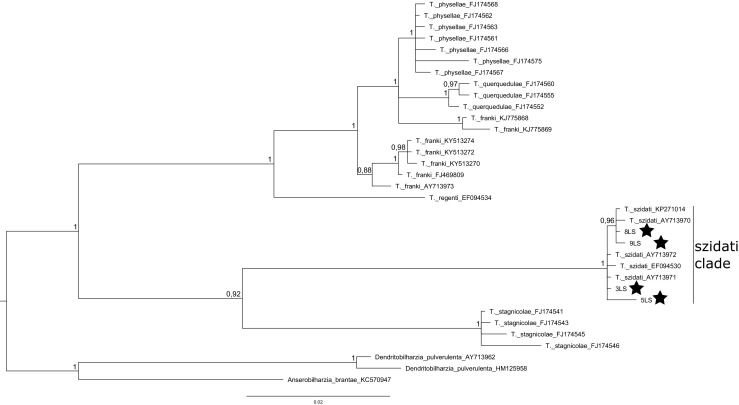
Bayesian phylogenetic tree of dataset X consisted of 34 sequences of bird schistosomes. Three sequences from representatives of *Dendrirobiharzia pulverulenta* and *Anserobiharzia brantae* were used as an outgroup. Numbers along the nodes are posterior probability of the node. DNA sequence revealed in this study is marked with a star

As a result of sequencing, we revealed four haplotypes for the ITS and one haplotype for the 28SrDNA (GenBank accession numbers: MH190224, MH190225, MH190226, MH190227, MH190228). Phylogenetic tree created with 28S rDNA (Fig. 2) (Table S4) showed that the one haplotype presented in this study belonged to *Trichobiharzia regenti*. Haplotype from our study, along with sequence of *T. regenti* possessed from GenBank NCBI, created one clade with the highest possible probability (100%) of the common node. Similarly, phylogenetic tree created for ITS (Fig. 3) (Table S5) allowed to determine species belonging of four revealed in this study haplotypes as *Trichobiharzia szidati.* All four haplotypes from this study connected with five sequences of *T. szidati* from GenBank and formed very well-supported (100% posterior probability) and distinct genetic clade.

### Seasonal fluctuations of Digenea larvae invasion in *L. stagnalis*

**Fig. 4:**
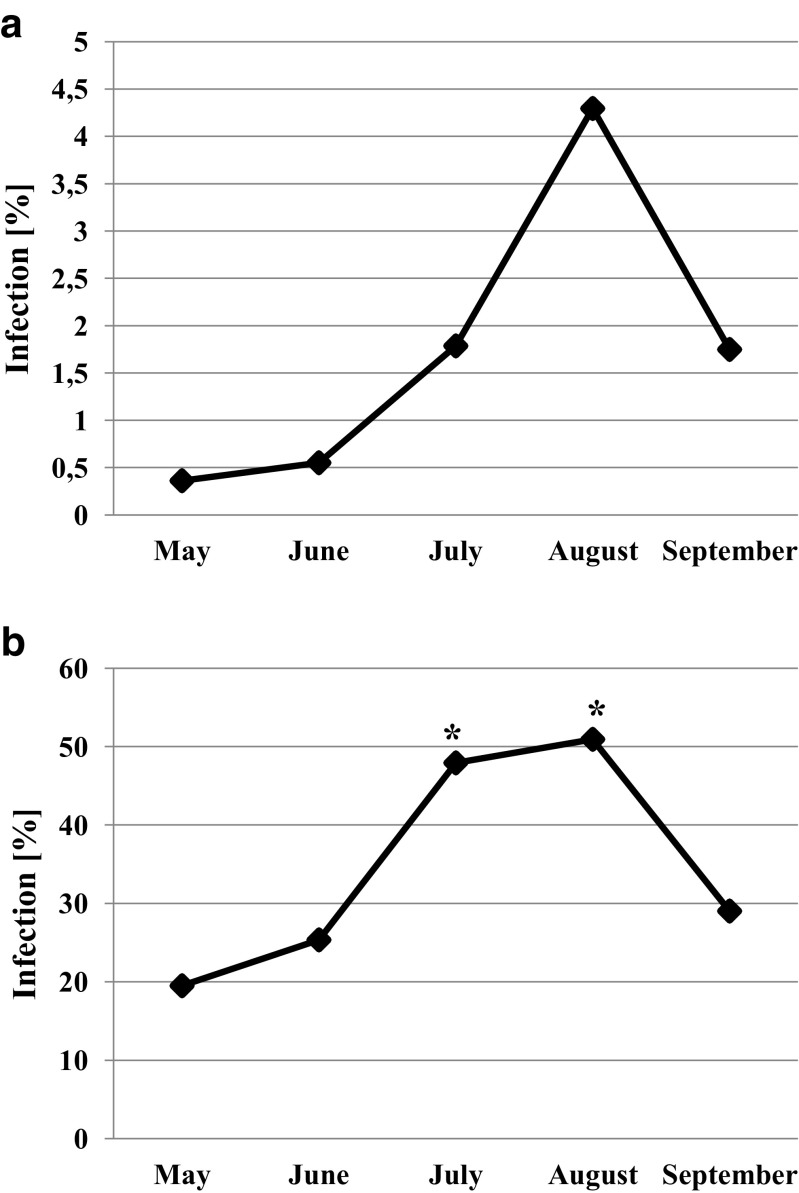
Seasonal infection of *Lymnaea stagnalis* with **a** *Trichobilharzia szidati* and **b** all digenean species in the growing season 2016. *Statistically different from May and June (post hoc Wilcoxon signed-rank test, *p* < 0.05)

Snails infected with bird schistosomes were found in all research months in 2016. Statistically significant seasonal fluctuations of schistosome infection were observed (*N* = 7, *χ*^2^ = 12.036, df = 4, *p* = 0.017) (Fig. 4a). A similar trend (Fig. 4b) is reflected in statistically significant differences for seasonal infection of all Digenea species found inside *L. stagnalis* individuals (N = 7, *χ*^2^ = 14.857, df = 4, *p* = 0.005). The post hoc test indicated that *L. stagnalis* infected with Digenea larvae was most frequently recorded in July and August.

### Infection of *L. stagnalis* with Digenea larvae related to four shell size classes

**Fig. 5:**
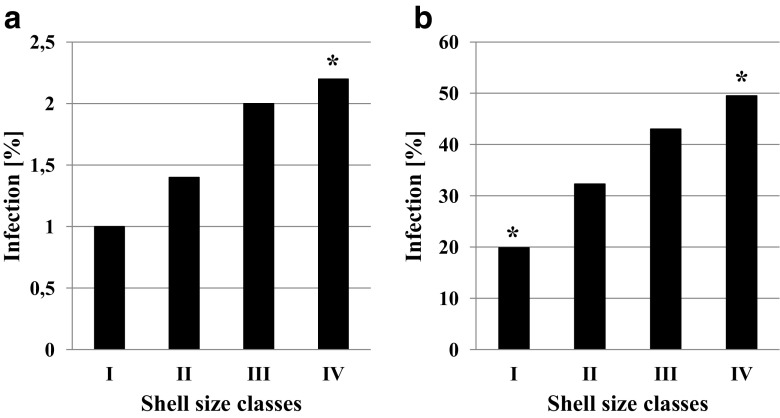
Infection of *Lymnaea stagnalis* of different shell size classes with **a** *Trichobilharzia szidati* and **b** all digenean species in the growing season 2016. Asterisks (*) indicate groups significantly contributing to the differences in the parasite presence (*z*-scores of standardized residuals)

Bird schistosomes were found in snails whose shell length ranged from 30.9 to 63.1 mm. We identified four shell size classes: class size I < 40.0 mm of shell length, class size II 40.1–44.9 mm, class size III 45.0–49.9 mm, and class size IV ≥ 50.0 mm. The number of bird schistosome hosts from individual size classes was significantly different (*χ*^2^ = 9.42, df = 3, *p* = 0.02). The post hoc test indicated that infected individuals of the highest shell size class were statistically significant. Similarly, the number of *L. stagnalis* infected with all Digenea (Fig. 5) was significantly dependent on correlated with shell size classes (*χ*^2^ = 119.83, df = 3, *p* < 0.001). Moreover, the post hoc test indicated that individuals from the highest and also lowest classes were statistically significant (Fig. 5).

## Discussion

Symptoms of swimmer’s itch caused by bird schistosomes depend greatly on their species. Since morphological and anatomical identifications of cercariae are unreliable (Dvorák et al. 2002; Horák et al. 2002; Rudolfová et al. 2005; Podhorský et al. 2009), human health risk assessment should be based on a molecular analysis of these larvae. Recent reports (Jouet et al. 2008, 2015; Christiansen et al. 2016) show much higher species diversity within the genus *Trichobilharzia* in Europe than previously thought. The following species have been recorded: *Trichobilharzia anseri* (Jouet et al. 2015), *Trichobilharzia franki* (Müller and Kimmig 1994), *Trichobilharzia mergi* (Kolářová et al. 2013b), *Trichobilharzia salmanticensis* (Simon-Martin and Simon-Vicente 1999), *T. regenti* (Horák et al. 1998), and *T. szidati* (Neuhaus 1952). Our study is the first to report the presence of *T. regenti* in Polish water bodies*.* The results also confirm widespread occurrence of *T. szidati* in Polish freshwater snails. What is surprising, despite the examination of nearly 1000 *Radix* sp. individuals, none of them was infected with *T. franki*—a bird schistosome widely distributed in the European populations of snail and waterfowl (Jouet et al. 2010). Both recorded species, as well as mentioned *T. franki* are widely recognized as causal agents of swimmer’s itch (Müller and Kimmig 1994; Żbikowska 2004). Bird schistosomes have been found in the majority of Polish lymnaeid species, but many reports have been limited to giving their complex name *T. ocellata* (Żbikowska 2004; Żbikowska et al. 2006; Cichy 2013). Only isolated cases of the presence of *T. szidati* in *L. stagnalis* (Żbikowska 2005) and *S. palustris* (Cichy 2013) as well as *T. franki* in *R. auricularia* (Żbikowska 2004) have been reported. In our previous study (Marszewska et al. 2016), we made an assumption that cercariae found in *R. balthica* belonged to the nasal bird schistosome species *T. regenti*. Molecular analysis performed in the present study confirmed this assumption. Also, the successful experimental infection of *R. balthica* by miracidia of *T. regenti* confirms the developing of this bird schistosome inside this species of *Radix* snails (Marszewska et al. 2018). However, the lack of molecular diagnosis of the *Radix* individuals naturally infected with *T. regenti* larvae does not allow to give the species name of hosts, that the conchological and anatomical data indicate that they were not snails belonging to the *R. auricularia* (Jackiewicz 2000; Piechocki and Wawrzyniak-Wydrowska 2016). For humans, the invasion of *T. regenti* may be more dangerous than that of other bird schistosomes. Nasal schistosomes migrate inside the final host through the nervous system (Kolářová et al. 2001; Leontovyč et al. 2016). Experimental studies have indicated that the movement of *T. regenti* within the nervous system of unusual mammalian hosts led to leg paralysis (Kouřilová et al. 2004; Lichtenbergová et al. 2011; Horák and Kolářová 2001). Although so far bird schistosomes have not been found inside the human body (Horák et al. 2015), it should be emphasized that in laboratory larvae of *Trichobilharzia* sp. invaded mammals through the skin and migrated to their internal organs (Horák and Kolářová 2001). In an experiment using mice, schistosomules of bird schistosomes were found in the lungs (Appleton and Brock 1986; Haas and Pietsch 1991; Horák and Kolářová 2000), heart, kidneys, liver, and intestines of these rodents (Haas and Pietsch 1991). According to Olivier (1953), they have also been found in the lungs of other mammalian hosts including hamsters, guinea pigs, rabbits, and even rhesus monkeys.

In Polish lakes, the prevalence of bird schistosomes was very low (Fig. 1), which corresponds to the observations of other European water bodies. The prevalence of *Trichobilharzia* spp. in intermediate hosts ranges from 0.05 to 5% (Soldánová et al. 2013) and is far lower than that of other Digenea. It should be noted that low prevalence of snails infected with these parasites does not exclude high risk of swimmer’s itch (Chamot et al. 1998; Lévesque et al. 2002; Farahnak and Essalat 2003; Skírnisson and Kolárová 2005; Jouet et al. 2008). This can be explained by very high bird schistosome cercarial emission (significantly higher than of other Digenean species) (Żbikowska 2005). Even low prevalence of invaded snails is sufficiently balanced by high intensity of cercarial release.

On the other hand, high risk of swimmer’s itch in European lakes results from seasonal fluctuations of the invasion. The results of our present and previous studies (Żbikowska 2004) confirm bird schistosome infection in snails collected from May to September, with the highest prevalence in the peak of the summer season (Fig. 4a). Therefore, risk assessment and preventive measures (e.g., removing snails from lakes) are extremely important (Chamot et al. 1998; Lévesque et al. 2002; Caumes et al. 2003; Verbrugge et al. 2004; Jouet et al. 2008).

Many authors have described seasonal changes in the prevalence of bird schistosomes and other Digenean species in snail hosts, indicating the highest rate during the warmest and the lowest during the coldest months (Loy and Haas 2001; Żbikowska et al. 2006; Żbikowska and Nowak 2009; Brown et al. 2010). This results from the fact that temperature has a huge impact on the life cycle of trematodes (Mas-Coma et al. 2009; Żbikowska and Cichy 2012). High temperature facilitates the transmission of parasites in the environment (Poulin 2006; Cichy et al. 2016) and stimulates the production of cercariae inside molluscs (Kendall and McCullough 1951; Lo and Lee 1996; Poulin 2006).

Finally, bird schistosome invasion inside host snails is an important factor affecting swimmer’s itch risk level. Our research shows that large snails are more often infected with flukes than small ones (Fig. 5), which is in line with the observations of other researchers (Loker 1983; Brown et al. 1988; Sorensen and Minchella 1998; Graham 2003; Sichun et al. 2005). According to Sichun et al. (2005), this correlation is beneficial for the parasite. Larger host snails provide greater energy resources and/or more space for the production of invasive cercariae (Graham 2003; Sichun et al. 2005). More intensive invasion of Digenea in snail hosts results in a bigger number of cercariae released into water and therefore with a higher risk of swimmer’s itch. The fact that larger snails are more often infected with these trematodes depends on many factors (Sturrock 1966; Baudoin 1975; Wilson and Denison 1980; McCarthy et al. 2004; Żbikowska et al. 2006; Miura and Chiba 2007). First of all, it may be connected to the preferences of parasites, which choose larger host snails over small ones (Baudoin 1975). On the other hand, it is well-know that the parasite may affect the host’s phenotypic traits, for example as the size of the shell (Miura and Chiba 2007). Scientists postulate that digenetic trematodes contribute to the abnormally large shells of host molluscs, known as parasitic gigantism (Wilson and Denison 1980; McCarthy et al. 2004; Żbikowska et al. 2006). However, when the snails are not yet infected, bigger snails are usually older snails and have more time to meet potentially a larger number of invasive larvae (Graham 2003; Sichun et al. 2005). Finally, smaller (younger) host snails are characterized by greater mortality because of the parasite (Sturrock 1966; Baudoin M; 1975) and as a result, smaller infected individuals are harder to find in the environment. This point of view is supported by our observation that large specimens of *L. stagnalis* invaded with bird schistosomes were collected in early May. Taking into account that the development of bird schistosomes from miracidia to cercariae takes about 7 weeks (Amen and Meuleman 1992), we can assume that snails releasing cercariae in May were invaded in autumn (McMullen and Beaver 1945; Jarcho and van Burkalow 1952) and survived winter (Horák et al. 2002). This situation is beneficial for bird schistosomes and increases the risk of swimmer’s itch, especially in the situation of recent climate change, and the earlier beginning of the recreational season in the temperate zone.

In conclusion, there are numerous indicators of real risk of swimmer’s itch including the following: (i) recent detection of the presence of potentially most dangerous nasal schistosome *T. regenti* in Poland, (ii) widespread presence of snails infected with *T. szidati* in Polish water bodies, (iii) widespread presence of these parasites during the summer season, (iv) frequent presence of these parasites in larger (more resistant) hosts. In view of these facts, we believe that it is necessary to develop effective methods of protection against cercarial dermatitis.

## Electronic supplementary material

ESM 1 (DOCX 16 kb)

ESM 2 (DOCX 16 kb)

ESM 3 (DOCX 18 kb)

ESM 4 (DOCX 13 kb)

ESM 5 (DOCX 14 kb)

ESM 6 (DOCX 15 kb)

ESM 7 (DOCX 15 kb)
