# Supplementary material for: Agents of swimmer’s itch—dangerous minority in the Digenea invasion of Lymnaeidae in water bodies and the first report of Trichobilharzia regenti in Poland
Source: Parasitol Res. 2018 Sep 13;117(12):3695–704. doi: 10.1007/s00436-018-6068-3 (PMC6224017; doi:10.1007/s00436-018-6068-3)
Supplement: Supplementary file 1 — (DOCX 16 kb) [file 436_2018_6068_MOESM1_ESM.docx]

Supplementary Material 1 Sequences of 28SrDNA possessed from GenBank NCBI and used in phylogenetic analysis along with accession number

| No. | Species | GenBank accession number |
| --- | --- | --- |
| 1 | *Trichobilharzia regenti* | AY157244.1 |
| 2 | *Trichobilharzia szidati* | AY157245.1 |
| 3 | *Trichobilharzia szidati* | FJ174476.1 |
| 4 | *Trichobilharzia physellae* | FJ174475.1 |
| 5 | *Trichobilharzia physellae* | FJ174474.1 |
| 6 | *Trichobilharzia physellae* | FJ174473.1 |
| 7 | *Trichobilharzia querquedulae* | FJ174468.1 |
| 8 | *Trichobilharzia querquedulae* | FJ174469.1 |
| 9 | *Trichobilharzia franki* | KJ775866.1 |
| 10 | *Trichobilharzia franki* | KJ775865.1 |
| 11 | *Trichobilharzia stagnicolae* | FJ174478.1 |
| 12 | *Trichobilharzia stagnicolae* | FJ174479.1 |
| 13 | *Trichobilharzia stagnicolae* | FJ174477.1 |
| 14 | *Dendritobilharzia pulverulenta* | AY157241.1 |
| 15 | *Gigantobilharzia huronensis* | AY157242.1 |
| 16 | *Trichobilharzia brantae* | FJ174466.1 |
| 17 | *Gigantobilharzia melanoidis* | JX875068.1 |
| 18 | *Allobilharzia visceralis* | EF114223.1 |
| 19 | *Allobilharzia visceralis* | EF114222.1 |
| 20 | *Anserobilharzia brantae* | KC570946.1 |
| 21 | *Trichobilharzia ocellata* | AF167089.1 |
| 22 | *Dendritobilharzia pulverulenta* | AF167090.1 |
| 23 | *Gigantobilharzia huronensis* | AF167091.1 |
| 24 | *Ornithobilharzia canaliculata* | AY157248.1 |
| 25 | *Austrobilharzia variglandis* | AY157250.1 |
| 26 | *Bivitellobilharzia loxodontae* | JN579949.1 |
| 27 | *Austrobilharzia terrigalensis* | AY157249.1 |
| 28 | *Schistosomatium douthitti* | AY157247.1 |
| 29 | *Heterobilharzia americana* | AY157246.1 |
| 30 | *Bivitellobilharzia nairi* | JQ975005.1 |
| 31 | *Austrobilharzia* sp. | JF742195.1 |
| 32 | *Bivitellobilharzia nairi* | AY858888.1 |
