# Supplementary material for: Agents of swimmer’s itch—dangerous minority in the Digenea invasion of Lymnaeidae in water bodies and the first report of Trichobilharzia regenti in Poland
Source: Parasitol Res. 2018 Sep 13;117(12):3695–704. doi: 10.1007/s00436-018-6068-3 (PMC6224017; doi:10.1007/s00436-018-6068-3)
Supplement: Supplementary file 3 — (DOCX 18 kb) [file 436_2018_6068_MOESM3_ESM.docx]

Table S1. Digenea prevalence [%] inside Lymnaea stagnalis

| Digenea species | Głuszyńskie^1^* | Ostrowąskie^1^ | Służewskie^1^ | Skulska wieś^1^ | Skulsk^1^ | Szymbarskie^1^ | Water Voley^1^ | Głuszyńskie^2^* | Skulska wieś^2^ | Skulsk^2^ | Sum^1. 2^ |
| --- | --- | --- | --- | --- | --- | --- | --- | --- | --- | --- | --- |
|  | P* [%] | P [%] | P [%] | P [%] | P [%] | P [%] | P [%] | P [%] | P [%] | P [%] | P [%] |
| Diplostomum pseudospathaceum | 5.80 | 8.14 | 11.11 | 12.03 | 10.58 | 0.77 | 26.67 | 11.41 | 6.88 | 9.30 | 9.94 |
| Echinoparyphium aconiatum | 1.45 | 12.79 | 15.28 | 2.29 | 0.00 | 2.07 | 9.05 | 0.00 | 1.38 | 1.16 | 4.17 |
| Echinostoma revolutum | 0.00 | 3.49 | 0.00 | 0.00 | 0.00 | 0.00 | 0.00 | 0.00 | 0.00 | 0.00 | 0.13 |
| Hypoderaeum conoideum | 0.00 | 0.00 | 2.08 | 0.00 | 0.68 | 0.52 | 2.38 | 0.00 | 0.00 | 0.00 | 0.65 |
| *Neoglyphe sobolevi* | 0.00 | 0.00 | 0.00 | 0.00 | 0.00 | 0.00 | 0.00 | 0.00 | 0.92 | 0.00 | 0.09 |
| Notocotylus attenuatus | 0.00 | 1.16 | 0.00 | 0.00 | 1.02 | 0.00 | 0.00 | 0.00 | 0.00 | 0.00 | 0.17 |
| Opisthoglyphe ranae | 23.91 | 1.16 | 0.35 | 5.73 | 9.90 | 11.11 | 0.00 | 8.15 | 4.59 | 9.88 | 7.27 |
| *Paryphostomum sp.* | 0.00 | 0.00 | 0.00 | 0.86 | 0.00 | 0.00 | 1.90 | 0.00 | 0.00 | 0.00 | 0.3 |
| Plagiorchis elegans | 5.80 | 2.32 | 6.25 | 5.73 | 3.75 | 2.07 | 1.90 | 5.98 | 0.46 | 13.37 | 4.56 |
| Sanguinicola inermis | 0.00 | 0.00 | 0.00 | 0.85 | 0.00 | 0.00 | 0.00 | 0.00 | 0.92 | 0.00 | 0.22 |
| Trichobilharzia sp. | 1.45 | 1.16 | 1.04 | 1.43 | 0.34 | 4.39 | 1.43 | 2.17 | 0.00 | 0.00 | 1.55 |
| Tylodelphys clavata | 0.00 | 2.33 | 0.35 | 0.00 | 0.00 | 0.00 | 0.48 | 0.00 | 0.00 | 0.58 | 0.22 |
| D. pseudospathaceum and O. ranae | 3.62 | 0.00 | 0.00 | 0.00 | 0.00 | 0.26 | 0.00 | 1.09 | 0.00 | 0.00 | 0.34 |
| D. pseudospathaceum and P. elegans | 0.72 | 0.00 | 0.00 | 0.00 | 0.00 | 0.00 | 0.00 | 0.00 | 0.00 | 0.00 | 0.04 |
| D. pseudospathaceum and Trichobilharzia sp. | 0.00 | 0.00 | 0.35 | 0.00 | 0.00 | 0.00 | 0.00 | 0.00 | 0.00 | 0.00 | 0.04 |
| O. ranae and P. elegans | 0.72 | 0.00 | 0.00 | 0.00 | 0.00 | 0.00 | 0.00 | 0.00 | 0.00 | 0.00 | 0.04 |
| O. ranae and S. inermis | 0.00 | 0.00 | 0.00 | 0.29 | 0.00 | 0.00 | 0.00 | 0.00 | 0.00 | 0.00 | 0.04 |
| O. ranae and Trichobilharzia sp. | 0.00 | 0.00 | 0.00 | 0.00 | 0.34 | 0.00 | 0.00 | 0.00 | 0.00 | 0.00 | 0.04 |
| Trichobilharzia sp. and undiagnosed pre-patent invasion | 0.00 | 0.00 | 0.00 | 0.00 | 0.00 | 0.00 | 0.48 | 0.00 | 0.00 | 0.00 | 0.04 |
| Undiagnosed pre-patent invasion | 5.07 | 3.50 | 4.86 | 3.44 | 5.80 | 2.84 | 11.90 | 9.24 | 10.55 | 10.46 | 6.32 |
| Sum | 48.55 | 36.05 | 41.67 | 32.66 | 32.76 | 24.03 | 56.67 | 38.59 | 26.15 | 44.77 | 36.34 |

^1^* research area in 2016; ^2^* research area in 2017; P* Digenea species prevalence
