# Supplementary material for: Agents of swimmer’s itch—dangerous minority in the Digenea invasion of Lymnaeidae in water bodies and the first report of Trichobilharzia regenti in Poland
Source: Parasitol Res. 2018 Sep 13;117(12):3695–704. doi: 10.1007/s00436-018-6068-3 (PMC6224017; doi:10.1007/s00436-018-6068-3)
Supplement: Supplementary file 4 — (DOCX 13 kb) [file 436_2018_6068_MOESM4_ESM.docx]

Table S2. Digenea prevalence [%] inside Stagnicola palustris

| Digenea species | Głuszyńskie^1^* | Ostrowąskie^1^ | Służewskie^1^ | Skulska wieś^1^ | Skulsk^1^ | Szymbarskie^1^ | Water Voley^1^ | Głuszyńskie^2^* | Skulska wieś^2^ | Skulsk^2^ | Sum^1, 2^ |
| --- | --- | --- | --- | --- | --- | --- | --- | --- | --- | --- | --- |
|  | P* [%] | P [%] | P [%] | P [%] | P [%] | P [%] | P [%] | P [%] | P [%] | P [%] | P [%] |
| Australapatemon burti | 0.00 | 0.00 | -* | 0.00 | 0.00 | - | 0.00 | 2.83 | - | 0.00 | 1.25 |
| Diplostomum pseudospathaceum | 0.00 | 0.00 | - | 9.09 | 0.00 | - | 9.43 | 6.60 | - | 50.00 | 5.83 |
| Echinoparyphium aconiatum | 0.00 | 11.11 | - | 18.18 | 0.00 | - | 3.77 | 0.00 | - | 0.00 | 2.08 |
| Hypoderaeum conoideum | 0.00 | 0.00 | - | 9.09 | 0.00 | - | 7.55 | 0.00 | - | 0.00 | 2.08 |
| Moliniella anceps | 0.00 | 0.00 | - | 9.09 | 19.05 | - | 0.00 | 0.00 | - | 0.00 | 2.08 |
| Opisthoglyphe ranae | 2.63 | 0.00 | - | 0.00 | 0.00 | - | 0.00 | 3.77 | - | 0.00 | 2.08 |
| Plagiorchis elegans | 0.00 | 0.00 | - | 0.00 | 0.00 | - | 0.00 | 0.94 | - | 0.00 | 0.42 |
| Undiagnosed pre-patent invasion | 0.00 | 0.00 | - | 9.09 | 14.29 | - | 1.89 | 7.55 | - | 0.00 | 5.42 |
| Sum | 2.63 | 11.11 | - | 54.54 | 33.33 | - | 22.64 | 21.70 | - | 50.00 | 21.25 |

^1^* research area in 2016; ^2^* research area in 2017; P* Digenea species prevalence; -* lack of *S. palustris* from the research area
