# Supplementary material for: Agents of swimmer’s itch—dangerous minority in the Digenea invasion of Lymnaeidae in water bodies and the first report of Trichobilharzia regenti in Poland
Source: Parasitol Res. 2018 Sep 13;117(12):3695–704. doi: 10.1007/s00436-018-6068-3 (PMC6224017; doi:10.1007/s00436-018-6068-3)
Supplement: Supplementary file 5 — (DOCX 14 kb) [file 436_2018_6068_MOESM5_ESM.docx]

Table S3. Digenea prevalence [%] inside *Radix* spp.

| Digenea species | Głuszyńskie^1^* | Ostrowąskie^1^ | Służewskie^1^ | Skulska wieś^1^ | Skulsk^1^ | Szymbarskie^1^ | Water Voley^1^ | Głuszyńskie^2^* | Skulska wieś^2^ | Skulsk^2^ | Sum^1. 2^ |
| --- | --- | --- | --- | --- | --- | --- | --- | --- | --- | --- | --- |
|  | P* [%] | P [%] | P [%] | P [%] | P [%] | P [%] | P [%] | P [%] | P [%] | P [%] | P [%] |
| Australapatemon burti | 3.33 | 0.00 | -* | 0.00 | 0.00 | 0.00 | 0.00 | 3.25 | 0.00 | - | 1.57 |
| *Cotylurus* sp. | 4.44 | 0.00 | - | 0.20 | 5.00 | 1.06 | 0.00 | 1.63 | 2.27 | - | 2.47 |
| Diplostomum pseudospathaceum | 0.56 | 22.22 | - | 0.05 | 0.00 | 0.00 | 33.33 | 0.81 | 9.09 | - | 1.24 |
| Echinoparyphium recurvatum | 2.22 | 0.00 | - | 0.20 | 5.00 | 4.25 | 33.33 | 0.81 | 2.27 | - | 2.25 |
| Hypoderaeum conoideum | 0.56 | 0.00 | - | 0.00 | 1.25 | 0.00 | 33.33 | 1.22 | 0.00 | - | 0.67 |
| Opisthoglyphe ranae | 7.22 | 0.00 | - | 0.20 | 1.25 | 1.06 | 0.00 | 4.88 | 2.27 | - | 3.60 |
| Plagiorchis elegans | 0.56 | 0.00 | - | 0.05 | 0.00 | 10.6 | 0.00 | 0.41 | 0.00 | - | 1.46 |
| Trichobilharzia sp. | 1.11 | 0.00 | - | 0.00 | 0.00 | 0.00 | 0.00 | 0.41 | 2.27 | - | 0.45 |
| *Tylodelphys clavata* | 0.00 | 0.00 | - | 0.30 | 0.00 | 0.00 | 0.00 | 0.00 | 0.00 | - | 0.67 |
| Undiagnosed pre-patent invasion | 5.00 | 11.11 | - | 0.15 | 5.00 | 1.06 | 0.00 | 2.85 | 6.82 | - | 3.15 |
| Sum | 27.78 | 33.33 | - | 1.15 | 17.50 | 18.09 | 10.00 | 16.26 | 25.00 | - | 18.09 |

^1^* research area in 2016; ^2^* research area in 2017; P* Digenea species prevalence; -* lack of *Radix* spp. from the research area
