# Supplementary material for: Agents of swimmer’s itch—dangerous minority in the Digenea invasion of Lymnaeidae in water bodies and the first report of Trichobilharzia regenti in Poland
Source: Parasitol Res. 2018 Sep 13;117(12):3695–704. doi: 10.1007/s00436-018-6068-3 (PMC6224017; doi:10.1007/s00436-018-6068-3)
Supplement: Supplementary file 7 — (DOCX 15 kb) [file 436_2018_6068_MOESM7_ESM.docx]

Table S5. Sequences of ITS possessed from GenBank NCBI and used in phylogenetic analysis along with accession number

| No. | Species | GenBank accession number |
| --- | --- | --- |
| 1 | *Trichobilharzia szidati* | AY713972 |
| 2 | *Trichobilharzia szidati* | KP271014 |
| 3 | *Trichobilharzia szidati* | EF094530 |
| 4 | *Trichobilharzia szidati* | AY713971 |
| 5 | *Trichobilharzia szidati* | AY713970 |
| 6 | *Trichobilharzia stagnicolae* | FJ174541 |
| 7 | *Trichobilharzia stagnicolae* | FJ174543 |
| 8 | *Trichobilharzia stagnicolae* | FJ174545 |
| 9 | *Trichobilharzia franki* | AY713973 |
| 10 | *Trichobilharzia querquedulae* | FJ174552 |
| 11 | *Trichobilharzia querquedulae* | FJ174560 |
| 12 | *Trichobilharzia franki* | FJ469809 |
| 13 | *Trichobilharzia stagnicolae* | FJ174546 |
| 14 | *Trichobilharzia querquedulae* | FJ174555 |
| 15 | *Trichobilharzia physellae* | FJ174568 |
| 16 | *Trichobilharzia physellae* | FJ174562 |
| 17 | *Trichobilharzia physellae* | FJ174563 |
| 18 | *Trichobilharzia physellae* | FJ174561 |
| 19 | *Trichobilharzia franki* | KY513274 |
| 20 | *Trichobilharzia franki* | KY513272 |
| 21 | *Trichobilharzia physellae* | FJ174566 |
| 22 | *Trichobilharzia franki* | KY513270 |
| 23 | *Trichobilharzia physellae* | FJ174575 |
| 24 | *Trichobilharzia physellae* | FJ174567 |
| 25 | *Trichobilharzia regenti* | EF094534 |
| 26 | *Trichobilharzia franki* | KJ775868 |
| 27 | *Trichobilharzia franki* | KJ775869 |
| 28 | *Dendritobilharzia pulverulenta* | AY713962 |
| 29 | *Anserobilharzia brantae* | KC570947 |
| 30 | *Dendritobilharzia pulverulenta* | HM125958 |
